# Effects of Exogenous Ethanol Treatment in Nutrient Solution on Growth and Secondary Metabolite Contents of Three Herb Species in an Indoor Vertical Farming System

**DOI:** 10.3390/plants12223842

**Published:** 2023-11-14

**Authors:** Juhyung Shin, YongJae Lee, Seungyong Hahm, Kwangya Lee, Jongseok Park

**Affiliations:** 1 Department of Bio-AI Convergence, Chungnam National University, Daejeon 34134, Republic of Korea; beetle304@naver.com (J.S.); zzcvxbcx51@naver.com (Y.L.); 2 Department of Horticultural Science, Chungnam National University, Daejeon 34134, Republic of Korea; seungyon96@naver.com; 3 Institute of Agricultural Science, Chungnam National University, Daejeon 34134, Republic of Korea

**Keywords:** hydroponics system, antioxidant activity, total phenolic contents, total flavonoids, growth parameter, herbaceous plants

## Abstract

This study aimed to explore the possibility of exogenous ethanol treatment as a technology to regulate the growth and the synthesis of secondary metabolites in herbaceous plants. After transplantation, sweet basil, Korean mint, and sweet wormwood were cultivated in a controlled vertical farming system and consistently exposed to exogenous ethanol at concentrations of 0, 0.5, 1, 2, 4, and 8 mM. Their growth parameters, antioxidant activity, and secondary metabolite contents were Everything is fine. measured to investigate the effects of the exogenous ethanol treatment on the three plants. The low-concentration ethanol treatments increased the shoot dry weight of the sweet basil and sweet wormwood compared to that of the control. As the ethanol concentration increased, the shoot fresh weight and leaf area in the sweet basil and Korean mint decreased compared to those of the control (0 mM). The DPPH (2,2-Diphenyl-1-picrylhydrazyl) radical scavenging activity and total phenolic content of the three plants increased with the ethanol concentration, while the total flavonoid content did not demonstrate a significant trend. The chlorophyll and carotenoids of the basil showed no apparent concentration-dependent trends; however, the chlorophyll and carotenoids of the Korean mint and sweet wormwood decreased with high ethanol concentrations. Moreover, the antioxidant enzyme activity increased with high ethanol concentrations, indicating that high ethanol concentrations induce oxidative stress in plants.

## 1. Introduction

Sweet basil (*Ocimum basilicum*), Korean mint (*Agastache rugosa*), and sweet wormwood (*Artemisia annua*) are annual herbs belonging to the category of herbaceous plants. Herbaceous plants produce organic compounds with unique properties as a defense mechanism against external factors. These compounds have been utilized by humans for centuries for various purposes such as medicinal and dietary uses [1]. The organic compounds of herbaceous plants are referred to as essential oils, and their compositions are largely derived from the terpenoid pathway, with aromatic amino acids serving as precursors from the phenylpropanoid pathway [2,3].

In recent years, there has been research aimed at devising various methods to enhance the production of these plant functional compounds and secondary metabolites [4]. Furthermore, exploration into innovative technologies is essential in agriculture and horticulture to improve plant production and quality. Techniques for regulating the synthesis of secondary metabolites offer opportunities to enhance crop yield, disease resistance, and stress tolerance in plants [5]. We aimed to explore the possibility of using exogenous ethanol treatment as a way to regulate the synthesis of secondary metabolites.

Ethanol (CH_3_CH_2_OH) is an organic compound formed by the combination of ethylene (C_2_H_4_) and water (H_2_O). It is widely used as a disinfectant, solvent, fuel, and more [6]. Moreover, it is recognized as one of the important compounds produced within plants in response to external environmental conditions. Ethanol is primarily generated by alcohol dehydrogenase (ADH) and serves various physiological and ecological functions within plants [7,8].

ADH enzymes exhibit high conservation across various plant species and play a critical role in their survival and stress responses [9,10,11,12]. Particularly, the high activity of ADH enzymes in various stress conditions suggests the potential application of exogenous ethanol in plant stress management [13,14]. Previous studies have provided evidence that ethanol can reversibly convert to acetyl-CoA and participate in respiration and biosynthesis through the PDH complex under anaerobic conditions [15,16]. However, depending on the ethanol concentration and exposure time, exceeding a certain range can cause cytotoxicity due to oxidative stress [17].

Research on the production of secondary metabolites in plants and the morphological effects of exogenous ethanol treatment under normal growth conditions is still limited. Therefore, this study aims to investigate the effects of exogenous ethanol treatment at various concentrations on the physiological and morphological changes of herbaceous plants under stress-free conditions. Through this study, we can expand our understanding of the physiological functions of exogenous ethanol and the stress response mechanisms in plants, which is expected to contribute to effective stress management and improvements in agricultural production.

## 2. Results

### 2.1. The Growth Parameter of Three Plants at Different Ethanol Concentrations

**Figure 1:**
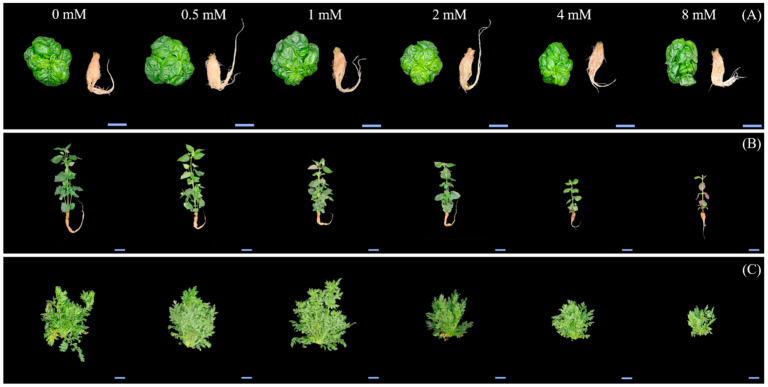
Representative images of sweet basil, Korean mint, and sweet wormwood grown in a hydroponics system at different ethanol concentrations (0, 0.5, 1, 2, 4, and 8 mM). Sweet basil and sweet wormwood were cultivated for 6 weeks, and Korean mint was cultivated for 4 weeks after transplantion. (**A**) sweet basil; (**B**) Korean mint; (**C**) sweet wormwood. The scale bars represent 10 cm.

**Figure 2:**
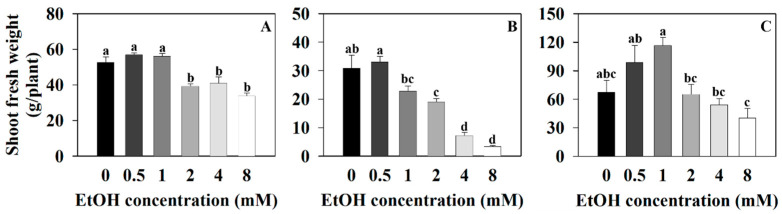
The shoot fresh weights of sweet basil, Korean mint, and sweet wormwood grown in a hydroponics system at different ethanol concentrations (0, 0.5, 1, 2, 4, and 8 mM). Sweet basil and sweet wormwood were cultivated for 6 weeks, and Korean mint was cultivated for 4 weeks after transplantion. (**A**) sweet basil; (**B**) Korean mint; (**C**) sweet wormwood. Data are represented as mean values ± standard error (*n* = 5). Different letters above the bars indicate a significant difference between treatments via Tukey’s HSD test at *p* ≤ 0.05.

Sweet basil and sweet wormwood were cultivated for 6 weeks, while Korean mint was cultivated for 4 weeks, and the representative images of plants grown in a hydroponics system at different ethanol concentrations are shown (Figure 1). Measurements of their shoot fresh weights, shoot dry weights, and leaf area were taken to analyze their growth at different ethanol concentrations. The shoot fresh weight of sweet basil was 52.48 g/plant for the control (0 mM) and 57, 56.16, 39.42, 41.14, and 33.92 g/plant for the 0.5, 1, 2, 4, and 8 mM ethanol treatments, respectively (Figure 2A). The shoot fresh weight of sweet basil decreased with the 2, 4, and 8 mM ethanol treatments compared to that of the control. The shoot fresh weight of Korean mint was 30.94 g/plant for the control and 33.08, 22.92, 19.12, 7.38, and 3.5 g/plant for the 0.5, 1, 2, 4, and 8 mM ethanol treatments, respectively (Figure 2B). The shoot fresh weight of Korean mint decreased with the 2, 4, and 8 mM ethanol treatments compared to that of the control. The shoot fresh weight of sweet wormwood was 67.69 g/plant for the control and 98.86, 116.62, 65.4, 54.64, and 40.7 g/plant for the 0.5, 1, 2, 4, and 8 mM ethanol treatments, respectively (Figure 2C). There were no significant differences among all the treatments, compared to the control.

**Figure 3:**
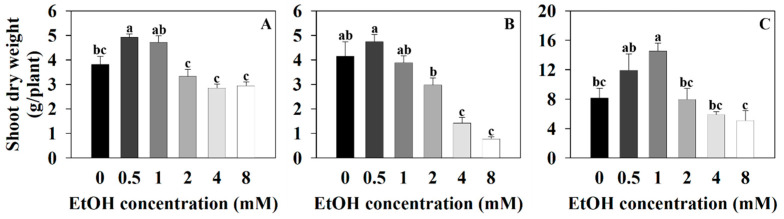
The shoot dry weights of sweet basil, Korean mint, and sweet wormwood grown in a hydroponics system at different ethanol concentrations (0, 0.5, 1, 2, 4, and 8 mM). Sweet basil and sweet wormwood were cultivated for 6 weeks, and Korean mint was cultivated for 4 weeks after transplantation. (**A**) sweet basil; (**B**) Korean mint; (**C**) sweet wormwood. Data are represented as mean values ± standard error (*n* = 5). Different letters above the bars indicate a significant difference between treatments via Tukey’s HSD test at *p* ≤ 0.05.

The shoot dry weight of sweet basil was 3.82 g/plant for the control (0 mM) and 4.92, 4.72, 3.33, 2.87, and 2.96 g/plant for the 0.5, 1, 2, 4, and 8 mM ethanol treatments, respectively (Figure 3A). The shoot dry weight of sweet basil increased with the 0.5 mM ethanol treatment compared to that of the control. The shoot dry weight of Korean mint was 4.15 g/plant for the control and 4.75, 3.9, 2.99, 1.44, and 0.8 g/plant for the 0.5, 1, 2, 4, and 8 mM ethanol treatments, respectively (Figure 3B). The shoot dry weight of Korean mint decreased with the 4 and 8 mM ethanol treatments compared to that of the control. The shoot dry weight of sweet wormwood was 8.19 g/plant for the control and 11.95, 14.55, 7.99, 5.92, and 5.14 g for the 0.5, 1, 2, 4, and 8 mM ethanol treatments, respectively (Figure 3C). The shoot dry weight of sweet wormwood increased with the 1 mM ethanol treatment compared to that of the control.

**Figure 4:**
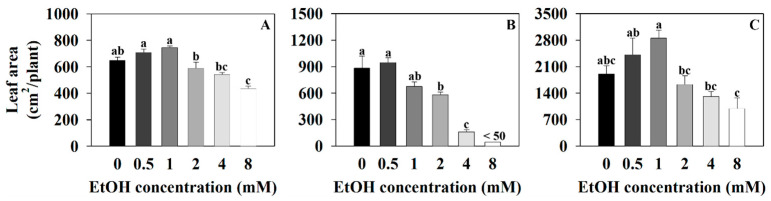
The leaf area of sweet basil, Korean mint, and sweet wormwood grown in a hydroponics system at different ethanol concentrations (0, 0.5, 1, 2, 4, and 8 mM). Sweet basil and sweet wormwood were cultivated for 6 weeks, and Korean mint was cultivated for 4 weeks after transplantation. (**A**), sweet basil; (**B**), Korean mint; (**C**), sweet wormwood. Data are represented as mean values ± standard error (*n* = 5). Different letters above the bars indicate a significant difference between treatments via Tukey’s HSD test at *p* ≤ 0.05.

The leaf area of sweet basil was 650.42 cm^2^/plant for the control (0 mM) and 650.42, 709.75, 744.7, 589.34, 543.26, and 437.59 cm^2^/plant for the 0.5, 1, 2, 4, and 8 mM ethanol treatments, respectively (Figure 4A). The leaf area of sweet basil decreased with the 8 mM ethanol treatment compared to that of the control. The leaf area of Korean mint was 884.59 cm^2^/plant for the control and 948.07, 678.62, 585.09, and 165.18 cm^2^/plant for the 0.5, 1, 2, and 4 mM ethanol treatments, respectively. It was less than 50 cm^2^/plant for the 8 mM ethanol treatment (Figure 4B). The leaf area of Korean mint decreased with the 2, 4, and 8 mM ethanol treatments compared to that of the control. The leaf area of sweet wormwood was 1910.7 cm^2^/plant for the control and 1910.7, 2416.4, 2854.51, 1633.69, 1315.9, and 1001.58 cm^2^/plant for the 0.5, 1, 2, 4, and 8 mM ethanol treatments, respectively (Figure 4C). There were no significant differences among all the treatments, compared to the control.

### 2.2. Antioxidant Capacity Measurements of Three Plants at Different Ethanol Concentrations

**Figure 5:**
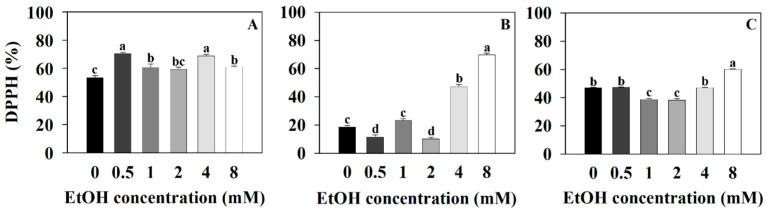
DPPH (2,2-diphenyl-1-picrylhydrazyl) radical scavenging activity of sweet basil, Korean mint, and sweet wormwood grown in a hydroponics system at different ethanol concentrations (0, 0.5, 1, 2, 4, and 8 mM). Sweet basil and sweet wormwood were cultivated for 6 weeks, and Korean mint was cultivated for 4 weeks after transplantation. (**A**) sweet basil; (**B**) Korean mint; (**C**) sweet wormwood. Data are represented as mean values ± standard error (*n* = 3). Different letters above the bars indicate a significant difference between treatments via Tukey’s HSD test at *p* ≤ 0.05.

Measurements of the DPPH (2,2-diphenyl-1-picrylhydrazyl) radical scavenging activity, SOD (superoxide dismutase) vigor, and POD (peroxidase) vigor were taken to analyze the plants’ antioxidant capacities at different ethanol concentrations. The DPPH radical scavenging activity of sweet basil was 53.4% for the control (0 mM) and 70.51, 60.58, 59.39, 68.8, and 60.92% for the 0.5, 1, 2, 4, and 8 mM ethanol treatments, respectively (Figure 5A). The DPPH radical scavenging activity of sweet basil increased with the 0.5, 1, 4, and 8 mM ethanol treatments compared to that of the control. The DPPH radical scavenging activity of Korean mint was 18.95% for the control and 11.9, 23.47, 10.54, 47.48, and 69.83% for the 0.5, 1, 2, 4, and 8 mM ethanol treatments, respectively (Figure 5B). The DPPH radical scavenging activity of Korean mint decreased with the 0.5 and 2 mM ethanol treatments and increased with the 4 and 8 mM ethanol treatments compared to that of the control. The DPPH radical scavenging activity of sweet wormwood was 47.14% for the control and 47.45, 38.91, 38.47, 47.24, and 60.2% for the 0.5, 1, 2, 4, and 8 mM ethanol treatments, respectively (Figure 5C). The DPPH radical scavenging activity of sweet wormwood decreased with the 1 and 2 mM ethanol treatments and increased with the 8 mM ethanol treatment compared to that of the control.

**Figure 6:**
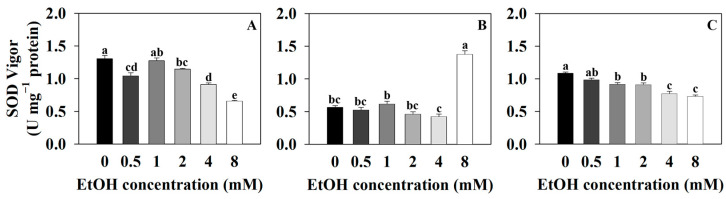
Superoxide dismutase (SOD) vigor of sweet basil, Korean mint, and sweet wormwood grown in a hydroponics system at different ethanol concentrations (0, 0.5, 1, 2, 4, and 8 mM). Sweet basil and sweet wormwood were cultivated for 6 weeks, and Korean mint was cultivated for 4 weeks after transplantation. (**A**) sweet basil; (**B**) Korean mint; (**C**) sweet wormwood. Data are represented as mean values ± standard error (*n* = 3). Different letters above the bars indicate a significant difference between treatments via Tukey’s HSD test at *p* ≤ 0.05.

The SOD vigor of sweet basil was 1.31 U·mg^−1^ protein for the control (0 mM) and 1.05, 1.28, 1.15, 0.92, and 0.66 U·mg^−1^ protein for the 0.5, 1, 2, 4, and 8 mM ethanol treatments, respectively (Figure 6A). The SOD vigor of sweet basil decreased with the 0.5, 2, 4, and 8 mM ethanol treatments compared to that of the control. The SOD vigor of Korean mint was 0.57 U·mg^−1^ protein for the control and 0.53, 0.62, 0.47, 0.43, and 1.38 U·mg^−1^ protein for the 0.5, 1, 2, 4, and 8 mM ethanol treatments, respectively (Figure 6B). The SOD vigor of Korean mint increased with the 8 mM ethanol treatment compared to that of the control. The SOD vigor of sweet wormwood was 1.09 U·mg^−1^ protein for the control and 0.99, 0.92, 0.91, 0.78, and 0.74 U·mg^−1^ protein for the 0.5, 1, 2, 4, and 8 mM ethanol treatments, respectively (Figure 6C). The SOD vigor of sweet wormwood decreased with 1, 2, 4, and 8 mM ethanol treatments compared to that of the control.

**Figure 7:**
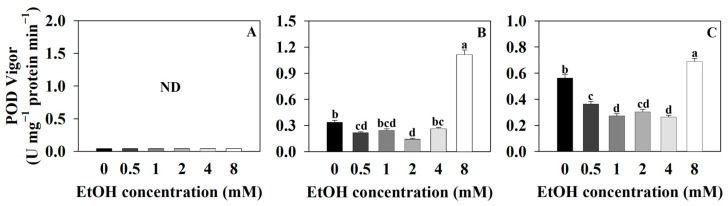
Peroxidase (POD) vigor of sweet basil, Korean mint, and sweet wormwood grown in a hydroponics system at different ethanol concentrations (0, 0.5, 1, 2, 4, and 8 mM). Sweet basil and sweet wormwood were cultivated for 6 weeks, and Korean mint was cultivated for 4 weeks after transplantation. (**A**) sweet basil; (**B**) Korean mint; (**C**) sweet wormwood. Data are represented as mean values ± standard error (*n* = 3). Different letters above the bars indicate a significant difference between treatments via Tukey’s HSD test at *p* ≤ 0.05 (ND, not detected).

In this study, the POD (peroxidase) vigor of sweet basil was not detected (Figure 7A). The POD vigor of Korean mint was 0.34 U·mg^−1^ protein·min^−1^ for the control and 0.22, 0.25, 0.15, 0.27, and 1.16 U·mg^−1^ protein·min^−1^ for the 0.5, 1, 2, 4, and 8 mM ethanol treatments, respectively (Figure 7B). The POD vigor of Korean mint decreased with the 0.5 and 2 mM ethanol treatments and increased with the 8 mM ethanol treatment compared to that of the control. The POD vigor of sweet wormwood was 0.56 U·mg^−1^ protein·min^−1^ for the control and 0.37, 0.28, 0.3, 0.27, and 0.69 U·mg^−1^ protein·min^−1^ for the 0.5, 1, 2, 4, and 8 mM ethanol treatments, respectively (Figure 7C). The POD vigor of sweet wormwood decreased with the 0.5, 1, 2, and 4 mM ethanol treatments and increased with the 8 mM ethanol treatment compared to that of the control.

### 2.3. The Secondary Metabolite Contents of Three Plants at Different Ethanol Concentrations

Measurements of the total chlorophyll (chlorophyll a + b), total carotenoid, total flavonoid, and total phenolic contents were taken to analyze the secondary metabolite content at different ethanol concentrations. The total chlorophyll content of sweet basil was 7.93 μg·mg^−1^ DW for the control (0 mM) and 6.87, 7.51, 8.27, 7.56, and 7.68 μg·mg^−1^ DW for the 0.5, 1, 2, 4, and 8 mM ethanol treatments, respectively (Table 1). The total chlorophyll content of sweet basil decreased with the 0.5 mM ethanol treatment compared to that of the control. The total chlorophyll content of Korean mint was 7.03 μg·mg^−1^ DW for the control and 6.53, 5.39, 6.41, 4.30, and 3.29 μg·mg^−1^ DW for the 0.5, 1, 2, 4, and 8 mM ethanol treatments, respectively (Table 1). The total chlorophyll content of Korean mint decreased with all the treatments compared to that of the control. The total chlorophyll content of sweet wormwood was 7.36 μg·mg^−1^ DW for the control and 7.86, 7.83, 8.19, 8.05, and 5.14 μg·mg^−1^ DW for the 0.5, 1, 2, 4, and 8 mM ethanol treatments, respectively (Table 1). The total chlorophyll content of sweet wormwood increased with the 2 mM ethanol treatment and decreased with the 8 mM ethanol treatment compared to that of the control.

The total carotenoid contents of sweet basil was 0.46 μg·mg^−1^ DW for the control (0 mM) and 0.38, 0.41, 0.38, 0.47, and 0.49 μg·mg^−1^ DW for the 0.5, 1, 2, 4, and 8 mM ethanol treatments, respectively (Table 1). There was no significant difference among all the treatments, compared to the control. The total carotenoid content of Korean mint was 0.65 μg·mg^−1^ DW for the control and 0.60, 0.47, 0.58, 0.43, and 0.28 μg·mg^−1^ DW for the 0.5, 1, 2, 4, and 8 mM ethanol treatments, respectively (Table 1). The total carotenoid content of Korean mint decreased with the 1, 2, 4, and 8 mM ethanol treatments, compared to that of the control. The total carotenoid content of sweet wormwood was 0.55 μg·mg^−1^ DW for the control and 0.60, 0.56, 0.65, 0.64, and 0.48 μg·mg^−1^ DW for the 0.5, 1, 2, 4, and 8 mM ethanol treatments, respectively (Table 1). The total carotenoid content of sweet wormwood decreased with the 8 mM ethanol treatment, compared to that of the control.

**Figure 8:**
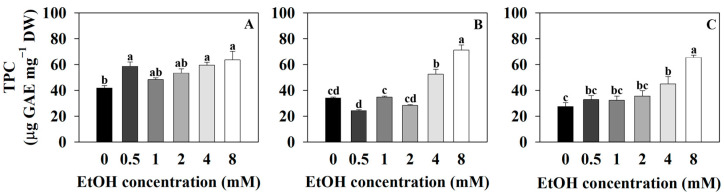
Total phenolic contents (TPC) of sweet basil, Korean mint, and sweet wormwood grown in a hydroponics system at different ethanol concentrations (0, 0.5, 1, 2, 4, and 8 mM). Sweet basil and sweet wormwood were cultivated for 6 weeks, and Korean mint was cultivated for 4 weeks after transplantation. (**A**) sweet basil; (**B**) Korean mint; (**C**) sweet wormwood. Data are represented as mean values ± standard error (*n* = 3). Different letters above the bars indicate a significant difference between treatments via Tukey’s HSD test at *p* ≤ 0.05.

The total phenolic content of sweet basil was 42.03 μg GAE·mg^−1^ DW for the control (0 mM) and 58.81, 48.55, 53.55, 59.66, and 63.74 μg GAE·mg^−1^ DW for the 0.5, 1, 2, 4, and 8 mM ethanol treatments, respectively (Figure 8A). The total phenolic content of sweet basil increased with the 0.5, 4, and 8 mM ethanol treatments, compared to that of the control. The total phenolic content of Korean mint was 34.22 μg GAE·mg^−1^ DW for the control and 24.49, 34.99, 28.54, 52.66, and 71.26 μg GAE·mg^−1^ DW for the 0.5, 1, 2, 4, and 8 mM ethanol treatments, respectively (Figure 8B). The total phenolic content of Korean mint increased with the 4 and 8 mM ethanol treatments, compared to that of the control. The total phenolic content of sweet wormwood was 27.7 μg GAE·mg^−1^ DW for the control and 33.04, 32.52, 35.75, 45.14, and 65.57 μg GAE·mg^−1^ DW for the 0.5, 1, 2, 4, and 8 mM ethanol treatments, respectively (Figure 8C). The total phenolic content of sweet wormwood increased with the 4 and 8 mM ethanol treatments compared to that of the control.

**Figure 9:**
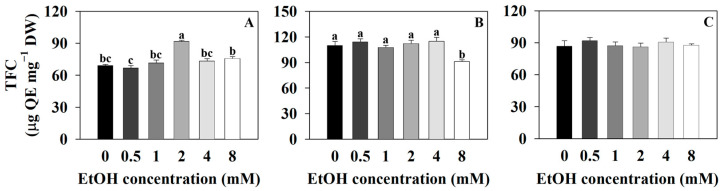
Total flavonoid content (TFC) of sweet basil, Korean mint, and sweet wormwood grown in a hydroponics system at different ethanol concentrations (0, 0.5, 1, 2, 4, and 8 mM). Sweet basil and sweet wormwood were cultivated for 6 weeks, and Korean mint was cultivated for 4 weeks after transplantation. (**A**) sweet basil; (**B**) Korean mint; (**C**) sweet wormwood. Data are represented as mean values ± standard error (*n* = 3). Different letters above the bars indicate a significant difference between treatments via Tukey’s HSD test at *p* ≤ 0.05.

The total flavonoid content of sweet basil was 69.3 μg QE·mg^−1^ DW for the control (0 mM) and 66.93, 71.73, 91.77, 73.53, and 75.92 μg QE·mg^−1^ DW for the 0.5, 1, 2, 4, and 8 mM ethanol treatments, respectively (Figure 9A). The total flavonoid content of sweet basil increased with the 2 mM ethanol treatment compared to that of the control. The total flavonoid content of Korean mint was 110.14 μg QE·mg^−1^ DW for the control and 114.39, 107.83, 112.29, 114.98, and 91.26 μg QE·mg^−1^ DW for the 0.5, 1, 2, 4, and 8 mM ethanol treatments, respectively (Figure 9B). The total flavonoid content of Korean mint decreased with the 8 mM ethanol treatment, compared to that of the control. The total flavonoid content of sweet wormwood was 86.84 μg QE·mg^−1^ DW for the control and 92.03, 87.37, 86.29, 90.7, and 87.86 μg QE·mg^−1^ DW for the 0.5, 1, 2, 4, and 8 mM ethanol treatments, respectively (Figure 9C). There were no significant differences among all the treatments, compared to the control.

## 3. Discussion

### 3.1. Ethanol Can Either Stimulate or Inhibit Cellular Respiration

The glucose within plant cells is converted into two pyruvate molecules through glycosis, and NAD^+^ is reduced to NADH. Under aerobic conditions, the pyruvate produces CO_2_ and ATP through the TCA (tricarboxylic acid) cycle inside the mitochondria, and NAD^+^ is reduced to NADH [18]. NADH is oxidized to NAD^+^ through the mitochondrial electron transport chain, producing ATP [19,20]. This series of processes occurring inside the mitochondria is referred to as “cellular respiration” [21]. NAD^+^ and NADH are coenzymes found in the mitochondria, and they play the roles of hydrogen acceptor and electron donor, respectively, in the TCA cycle and electron transport chain [22,23]. They are essential components in cellular respiration. ATP serves as the energy source for the cell and is consumed to support various life processes [24].

Ethanol can influence the carbon balance through the PDH (pyruvate dehydrogenase) bypass pathway. The PDH bypass is known to contribute to energy and biosynthesis in yeast [25]. ADH (alcohol dehydrogenase) and ALDH (aldehyde dehydrogenase), which are involved in this process, exhibit high homology in eukaryotes, and previous studies provide clear evidence that the PDH bypass plays a similar role in plants [26,27]. When ^14^C-labeled ethanol was supplied to tobacco pollen tubes and the metabolism was analyzed, it was confirmed that most of it was detected in the form of CO_2_ and lipids, indicating its involvement in cellular respiration and lipid biosynthesis [28]. Moreover, a significant number of the detected lipids were identified as phospholipids, which play a structurally important role within cells [29]. In anaerobic conditions, ethanol and NAD^+^ can be catalyzed by ADH and ALDH, along with CoA, converting them into acetyl-CoA (coenzyme) and NADH [30,31,32]. Acetyl-CoA can be utilized not only in lipid biosynthesis but also in the production of citrate within the mitochondrial TCA cycle.

In a previous study, when tomato (*Lycopersicon esculentum*) plants were subjected to foliar treatment with 15% and 20% alcohol (methanol and ethanol) compared to an untreated control, their stem fresh weight and dry weight increased by 22% and 31% and their leaf fresh weight and dry weight increased by 19% and 17%, respectively [33]. When ethanol was applied to the leaves or roots of strawberry (*Fragaria ananasa* ‘Gaviota’) plants, there was an increase in their fruit weight and yield compared to those of the untreated control [34]. When 20 mM ethanol was applied to soybean (*Glycine max*) plants, the plant height and shoot dry weight increased 12.35% and 32.38%, respectively, compared to those of an untreated control [13]. These research results suggest that exogenous ethanol can have an impact on the physiological activities or growth promotion of plant organisms.

In this study, the shoot fresh weight and leaf area of basil and Korean mint decreased as the ethanol concentration increased compared to those of the control (Figure 2B and Figure 4B). However, the shoot dry weight of basil increased with the 0.5 mM ethanol treatment compared to that of the control, and the shoot dry weight of sweet wormwood increased with the 1 mM ethanol treatment compared to that of the control (Figure 3A,C). This suggests that the physiological and morphological responses to exogenous ethanol treatment can vary depending on the species. As absorbed by the roots, exogenous ethanol can contribute to the carbon balance and potentially enhance plant growth.

High intracellular levels of NADH can inhibit the action of isocitrate dehydrogenase in the citric acid cycle and alpha-ketoglutarate dehydrogenase, thereby suppressing the processing of acetyl-CoA and reducing cellular respiration [35,36]. Also, acetaldehyde can induce DNA damage and oxidative stress within cells, which may decrease growth promotion [37,38,39]. In this study, the 2, 4, and 8 mM ethanol treatments led to reduced cellular respiration due to the accumulation of acetaldehyde, resulting in oxidative stress and the functional loss of mitochondria. This is caused a decrease in the shoot fresh weight, dry weight, and leaf area (Figure 2, Figure 3 and Figure 4).

### 3.2. High-Concentration Ethanol Treatment in Plants Induces Oxidative Stress

High concentrations of ethanol induce various stresses in mitochondria. These include an increase in mitochondrial ROS, the leakage of amino acids and nucleotides, functional losses due to protein structural changes, the disruption of the mitochondrial membrane potential leading to impaired proton transport, and interference with the cell structure, among others. High concentrations of ethanol can lead to the functional impairment of mitochondria, resulting in various physiological changes and growth inhibition [40]. In this study, the DPPH radical scavenging activity, POD vigor, and SOD vigor were measured to evaluate the extent of ROS generated by ethanol.

DPPH (2,2-diphenyl-1-picrylhydrazyl) is a molecule with a nitrogen-centered free radical that exists in a stabilized state [41]. DPPH can eliminate ROS (reactive oxygen species) through the hydrogen atom transfer (HAT) and single electron transfer (SET) mechanisms, and DPPH radical scavenging activity can be used to evaluate antioxidant activity [42]. The DPPH radical scavenging activity of all plants increased with the 8 mM ethanol treatment compared to that of the control, and the results show that oxidative stress occurred with the 8 mM ethanol treatment (Figure 5). The 0.5, 1, 2, and 4 mM ethanol treatments resulted in different DPPH radical scavenging activities, indicating that the oxidative stress levels induced by different ethanol concentrations may vary for each plant (Figure 5).

Superoxide is one of the free radicals that is generated by the removal of one electron from an oxygen molecule, and it is produced during cellular respiration and various biological reactions in the organism, leading to oxidative stress within cells [43,44]. SOD (superoxide dismutase) oxidizes superoxide to produce H_2_O_2_ or O_2_, reducing oxidative stress within the cell [45,46]. H_2_O_2_ acts as an intermediate in chemical reactions related to oxidative stress and is generated as a byproduct of cellular activities within the cell [47,48]. POD (peroxidase) oxidizes H_2_O_2_ to produce H_2_O and O_2_, thereby suppressing oxidative stress by eliminating intermediates of oxidative stress [49,50]. Therefore, it is suggested that as the SOD vigor increases, the superoxide will be oxidized, leading to an increase in H_2_O_2_. The increased H_2_O_2_ can then be oxidized by the POD.

The SOD vigor of sweet basil and sweet wormwood decreased as the ethanol concentration increased (Figure 6A,C). The increased DPPH radical scavenging activity of sweet basil and sweet wormwood indicates that the oxidative stress increased with the 4 and 8 mM ethanol treatments compared to that of the control (Figure 5A,C). It is suggested that the ROS generated by the ethanol treatment was not superoxide in the sweet basil and sweet wormwood. The SOD vigor of Korean mint increased with the 8 mM ethanol treatment compared to that of the control. It is indicated that superoxide was generated with the 8 mM ethanol treatment (Figure 6B). As the SOD vigor of Korean mint increased, the superoxide was oxidized to produce H_2_O_2_ with the 8 mM ethanol treatment. Consequently, it is suggested that the POD vigor also increased as the H_2_O_2_ increased (Figure 7B).

### 3.3. Effect of Exogenous Ethanol Treatment in Plants on Secondary Metabolite Contents

Oxidative stress occurs when reactive oxygen species (ROS), which possess electrons, oxidize DNA, proteins, and lipids within plant cells, leading to the inhibition of plant growth [51,52]. Chlorophyll increases in conditions in which ROS are generated and its activity can eliminate ROS [53,54]. Furthermore, chlorophyll and carotenoids serve as photosynthetic pigments, collecting light in the photosystem and playing an essential role in the photosynthetic process [55,56]. The 2 mM ethanol treatment did not affect the growth of sweet wormwood but increased the total chlorophyll content (Figure 1C, Table 1) compared to that of the control. The increased photosynthetic pigments enhance the photosynthetic rates and the production of photosynthetic products [57]. Therefore, the 2 mM ethanol treatment in sweet wormwood is expected to help increase the production of photosynthetic products as its chlorophyll content rises, its survival under adverse environments, or in reducing oxidative stress.

Exogenous ethanol treatment in Arabidopsis thaliana was found to be involved in phenylpropanoid biosynthesis [58]. Phenylpropanoids originate from phenylalanine and can be transformed into various phenolic compounds, lignins, and flavonoids through interactions with several enzymes [59]. Lignin forms the cell wall structure, while phenols and flavonoids, acting as peroxidase (POD) within plant cells, can convert H_2_O_2_ into H_2_O, thus reducing oxidative stress [60,61,62,63]. The contents of phenols and flavonoids increased in environments in which oxidative stress occurred, such as in drought and cold stress conditions [64,65]. Previous research suggests that the contents of phenols and flavonoids within plants can increase under oxidative stress conditions as a defense mechanism. Hence, exogenous ethanol treatment in plants induces oxidative stress, and since oxidative stress is linked to the precursor of phenols and flavonoids, phenylpropanoid, it is anticipated that the total phenol and flavonoid contents will change.

In the three plants, as the ethanol concentration increased, the total phenolic contents increased (Figure 8). The 8 mM ethanol treatment resulted in an increased DPPH radical scavenging activity and POD (peroxidase) vigor compared to those of the control (Figure 6 and Figure 8). The 8 mM ethanol treatment is considered to have induced oxidative stress in the three plants, leading to increased total phenolic contents as a defense mechanism to reduce oxidative stress. The varying total phenolic contents observed with the 0.5, 1, 2, and 4 mM ethanol treatments in each plant suggest that the oxidative stress responsiveness differ among these plants (Figure 8). The flavonoid pathway begins with the conversion of p-coumaroyl-CoA to chalcone, catalyzed by malonyl CoA and chalcone synthase. Subsequently, chalcone is oxidized by enzymes to produce various flavonoid compounds [66]. When the leaves of soybean (*Glycine max*) plants were treated with a 20 mM ethanol spray, there was no change in total flavonoid contents compared to those of the control [13]. In this study, as the ethanol concentration increased, there was no significant trend observed in the change in total flavonoid contents (Figure 9). This suggests that exogenous ethanol treatment does not have an impact on flavonoid pathway in plants.

## 4. Materials and Methods

### 4.1. Plant Materials and Environmental Conditions of the Seedlings

Sweet basil, Korean mint, and sweet wormwood seeds were sown in 128-cell trays with rockwool cubes (2.5 × 2.5 × 4 cm, Grodan, Roermond, The Netherlands). The seeds were sown and subjected to a 48-h dark treatment at a temperature of 22 ± 2 °C in a controlled vertical farming system (Daejeon, Republic of Korea (36°22′11″ N 127°21′10″ E, elevation = 60 m). Subsequently, they were grown for 3 weeks under the following growth conditions: 150 ± 10 μmol·m^−2^·s^−1^ light intensity, a relative humidity of 70–75%, and a photoperiod of 16/8 h (light/dark). Beginning with the appearance of foliage leaves, the plants were irrigated every other day with Hoagland solution composed of N 14.6 me·L^−1^, P 1 me·L^−1^, K 6 me·L^−1^, Ca 7.6 me·L^−1^, Mg 4 me·L^−1^, and S 4 me·L^−1^. The solution was adjusted to a pH of 6.5 ± 0.3 and an EC (electric conductivity) of 0.8 ± 0.2 dS·m^−1^.

### 4.2. Environment Conditions and Ethanol Treatments after Transplantation

Three weeks after sowing, uniform 15 seedlings were selected and transplanted into a semi-DFT (deep-flow technique) hydroponic system in a controlled vertical farming system for each treatment. The plants were grown under the following environmental conditions: a temperature of 25/20 ± 1 °C, a photoperiod of 16/8 h (light/dark), relative humidity of 70 ± 10%, and a light intensity of 200 ± 10 μmol·m^−2^·s^−1^. Sweet basil and sweet wormwood were cultivated for 6 weeks, and Korean mint was cultivated for 4 weeks in the semi-DFT hydroponic system. Hoagland solution with a pH of 6.5 ± 0.3 and an EC of 2.0 ± 0.2 dS·m^−1^ was mixed with ethanol at concentrations of 0, 0.5, 1, 2, 4, and 8 mM for the respective plants during the cultivation period. The Hoagland solution and ethanol were replaced at one-week intervals to maintain the balance of nutrients.

### 4.3. Measurements of Plant Growth Parameters

Sweet basil, Korean mint, and sweet wormwood were harvested from five plants per treatment, and measurements were taken to analyze shoot fresh weight, shoot dry weight, and leaf area. Shoot fresh weight and dry weight were measured using an electronic balance (MW-2N, CAS Co. Ltd., Yangju, Republic of Korea), while leaf area was measured using a leaf area meter (LI-3100, LI-COR Co., Lincoln, NE, USA). The samples were dried in a drying oven (HB-501M, Hanbaek Scientific Technology Co., Ltd., Bucheon, Republic of Korea) set at 70 °C for 7 days. Subsequently, the shoot dry weight was measured using an electronic balance.

### 4.4. Preparation of the Extract

Sweet basil, Korean mint, and sweet wormwood were harvested in three plants per treatment to measure secondary metabolite contents. Chlorophyll a and b content, total carotenoids, total phenolic contents, and total flavonoid contents were determined. The harvested plant material was rapidly frozen using liquid nitrogen and stored at −75 °C in a deep freezer (ULT-387CR, GMS, Dongducheon, Republic of Korea). Frozen samples were freeze-dried using a freeze dryer (TFD550, Ilshin BioBase Co. Ltd., Dongducheon, Republic of Korea) and then powdered. Powdered samples (20 mg) were mixed with 2 mL of 90% H_2_O-methanol solvent using a Voltex Mixer (SI-0246A, Coleparmer, Vernon Hills, IL, USA). Subsequently, the mixture was sonicated at 40 °C for 40 min using an Ultrasonic Bath (powersonic420, Hwashin Tech Co., Ltd., Yeongcheon, Republic of Korea) and then centrifuged at 21,092× *g* for 5 min (Smart 15 plus, Hanil, Seoul, Republic of Korea). The resulting supernatant was used for measuring chlorophyll pigment content, total phenolic content, total flavonoid content, and DPPH radical scavenging ability.

### 4.5. Extraction of Enzymes

The following procedure was conducted to prepare enzyme extracts for the measurement of total soluble protein, superoxide dismutase (SOD), and guaiacol peroxidase (POD), maintaining a cold environment throughout. Powdered samples (20 mg) were mixed with 50 mM sodium phosphate buffer (2 mL) at a pH of 7.0, and cell lysis was carried out by subjecting the mixture to three cycles of freezing and thawing using liquid nitrogen and ice/water, as described by [67]. Subsequently, the mixture was centrifuged at 21,092× *g* for 5 min at 4 °C, and the supernatant was separated and used for analysis.

### 4.6. Chlorophyll Pigments and Total Carotenoids

Chlorophyll a, chlorophyll b, and total carotenoid content were measured by modifying the method of [68]. The prepared sample extract was dispensed (200 μL) into a 96-well plate (SPL30096, SPL., Republic of Korea), and absorbance was measured at 470 nm, 652 nm, and 665 nm using a microplate spectrophotometer (EpochTM, Agilent Technologies, Santa Clara, CA, USA). The contents of chlorophyll a, chlorophyll b, and total carotenoids per unit dry weight were expressed in μg·mg^−1^ DW based on the following calculations (*n* = 3).

$$c_{a}\;(\mu g/mL)\;=\;16.82\;A_{665.2\;}-\;9.28\;A_{652.4}$$

$$c_{b}\;(\mu g/mL)\;=\;36.92\;A_{652.4}\;-\;16.54\;A_{665.2}$$

$$c_{\left(x+c\right)}\;(\mu g/mL)\;=\;(1000\;A_{470}\;-\;1.91\;c_{a}\;-\;95.15\;c_{b})/225$$

$c_{a}$: Chlorophyll a

$c_{b}$: Chlorophyll b

$c_{\left(x+c\right)}$: Carotenoids (x + c = xanthophylls and carotenes)

### 4.7. Total Phenolic Contents

Total phenolic contents were measured by modifying the method of [69]. The samples were the supernatant previously used for chlorophyll measurement. The supernatant or standard substance (75 μL), Folin & Ciocalteu’s phenol reagent (F9252, Sigma-Aldrich, St. Louis, MO, USA) (75 μL), and distilled water (1125 μL) were mixed and allowed to react for 5 min. Then, a 7.5% Na_2_CO_3_ solution (225 μL) was added, and the mixture was allowed to react at room temperature for 40 min. Subsequently, 200 μL of the mixture was dispensed, and absorbance was measured at 765 nm. The measured values were substituted into a standard curve using gallic acid to calculate the equivalent value, and the total phenolic contents per unit dry weight were expressed in μg GAE·mg^−1^ DW (*n* = 3).

### 4.8. Total Flavonoid Contents

Total flavonoid contents were measured by modifying the method of [70]. The sample (100 μL) was mixed with 95% ethanol (300 μL), 10% aluminum chloride (20 μL), 1M potassium acetate (20 μL), and distilled water (600 μL) and allowed to react at room temperature for 40 min. The reacted mixture was then measured for absorbance at 415 nm. The measured values were substituted into a standard curve using quercetin to calculate the equivalent value, and the total flavonoid contents per unit dry weight were expressed in μg QE·mg^−1^ DW (*n* = 3).

### 4.9. DPPH Radical Scavenging Assay

DPPH radical scavenging ability was measured by appropriately modifying the method of [71]. A DPPH solution was prepared by mixing 2,2-Diphenyl-1-picrylhydrazyl (D9132, Sigma-Aldrich, USA) (200 mg) with MeOH (50 mL). A volume of 170 μL 90% MeOH, DPPH solution (10 μL), and the sample (20 μL) were mixed and allowed to react for 30 min in a darkroom. Absorbance was then measured at 517 nm. The control was prepared without the sample, and the DPPH radical scavenging rate (%) was calculated by substituting the values into the following formula (*n* = 3).

$$DPPH\;radical\;scavenging\;rate\;sample\;\left(\%\right)=\frac{A517\;control-A517\;sample}{A517\;control}\times 100\%$$

### 4.10. Total Soluble Protein

Total soluble protein content was quantified using [72]’s method. The prepared enzyme extract (4 μL) was mixed well with Bradford’s reagent (196 μL) and allowed to react for 20 min. The reacted mixture was then measured for absorbance at 565 nm. The measured values were substituted into a standard curve using bovine serum albumin (A5611, Sigma-Aldrich, USA) to calculate the equivalent value. The soluble protein content per unit dry weight was expressed in μg·mg^−1^ DW, and this value was used to calculate the total enzyme activity per unit protein for peroxidase (POD) and superoxide dismutase (SOD).

### 4.11. SOD Activity (EC 1.15.1.1)

Superoxide dismutase (SOD) activity was measured by appropriately modifying the method of [50]. A reaction mixture was prepared by thoroughly mixing 50 mM at a pH of 7.0, sodium phosphate (93.5 μL), 0.1 M methionine (52 μL), 2.5 mM NBT (24.5 μL), 10 mM EDTA (2 μL), and 0.5 mM riboflavin (8 μL). The control, without enzyme extract, was exposed to PPFD (photosynthetic photon flux density) of 50 μmol·m^−2^·s^−1^ LED light simultaneously with the samples for 15 min, followed by light blocking. Absorbance was then measured at 560 nm, and SOD activity was expressed in units of enzyme activity per milligram of dry weight (unit mg^−1^ DW) using the formula below, with unit defined as the amount of enzyme that caused a 50% reduction in NBT reduction rate (*n* = 3). The Blank, which contained no enzyme extract, was stored in the dark, and absorbance was measured to confirm the establishment of temperature equilibrium.

$$SOD\;inhibition\;\left(\%\right)=\frac{A560\;control-A560\;enzyme}{A560\;control}\times 100\%$$

$$SOD\;activity\;\left(unit\;{mL}^{-1}\right)=\frac{SOD\;inhibition\times total\;volume}{50\times enzyme\;volume}$$

$$SOD\;activity\;\left(unit\right)=\frac{unit\;{mL}^{-1}}{enzyme\;({mg\;mL}^{-1})}$$

### 4.12. POD Activity (EC 1.11.1.7)

Peroxidase (POD) activity was measured by appropriately modifying the method of [73]. In the reaction mixminture prepared by mixing 40 mM sodium phosphate buffer at a pH of 6.1 (66.6 μL), 20 mM guaiacol (80 μL), and 3% H_2_O_2_ (33.3 μL), enzyme extract (20 μL) was added. Absorbance was measured at 470 nm every 10 s, and POD activity was calculated in µmol min^−1^·mg^−1^ DW using the formula below (*n* = 3). The Blank was prepared without enzyme extract in the reaction mixture to confirm the achievement of temperature equilibrium based on absorbance measurements.

$$POD\;activity\;\left(µmol\;{min}^{-1}{mL}^{-1}\right)=\frac{(A470/min)\times total\;volume\times 1000}{26.6\times enzyme\;volume}$$

$$POD\;activity\;\left(µmol\;{min}^{-1}{mg}^{-1}DW\right)=\frac{unit\;{mL}^{-1}}{enzyme\;(mg\;{mL}^{-1})}$$

The wavelength for absorption reading was 470 nm for 1 min, and the extinction coefficient was 26.6 mM^−1^·cm^−1^.

### 4.13. Statistical Analyses

Data were statistically analyzed using analysis of variance (ANOVA) test with Tukey’s HSD (honestly significant difference) test for a significance of *p* ≤ 0.05 using the SPSS program (SPSS 26, SPSS Inc., Chicago, IL, USA). Graphs were produced using Sigmaplot (15.0, Systat Software, Inc., Chicago, IL, USA).

## 5. Conclusions

In this study, low-concentration ethanol treatments increased the shoot dry weight of sweet basil and sweet wormwood compared to that of the control. It is suggested that the exogenous ethanol was converted into acetyl-CoA by ADH and ALDH enzymes in the plants, with pyruvate utilized in the mitochondrial TCA cycle to increase their ATP production. The increased ATP was used in the Calvin cycle, leading to increased biosynthetic products. The total chlorophyll and carotenoids exhibited different trends in each plant. In the three plants, the DPPH radical scavenging activity increased with the 8 mM ethanol treatment compared to that of the control. This indicates that the high-concentration ethanol treatment induced oxidative stress in the plants, leading to increased total phenolic contents as a defense mechanism in the plants. The exogenous ethanol treatment is considered not to have affected the flavonoid pathway, and this study did not observe any specific trends in terms of the total flavonoid content. Therefore, low-concentration ethanol treatments can be expected to increase plants’ shoot dry weight, while high-concentration ethanol treatments are considered to induce strong oxidative stress within plants.

## Figures and Tables

**Table 1:** The photosynthetic pigments of sweet basil, Korean mint, and sweet wormwood at different ethanol concentrations (0, 0.5, 1, 2, 4, and 8 mM). Sweet basil and sweet wormwood were cultivated for 6 weeks, and Korean mint was cultivated for 4 weeks after transplantation.

Plant Name	Treatment (mM)	Photosynthetic Pigments (μg·mg^−1^ DW)			
		Chlorophyll a	Chlorophyll b	Chlorophyll a + b	Carotenoids
Sweet basil	0	5.12 ± 0.16 a	2.81 ± 0.08 b	7.93 ± 0.24 a	0.46 ± 0.02 ab
	0.5	4.39 ± 0.10 b	2.48 ± 0.04 c	6.87 ± 0.14 b	0.38 ± 0.01 b
	1	4.76 ± 0.12 ab	2.75 ± 0.04 b	7.51 ± 0.15 ab	0.41 ± 0.02 ab
	2	5.12 ± 0.13 a	3.14 ± 0.05 a	8.27 ± 0.18 a	0.38 ± 0.02 b
	4	4.88 ± 0.07 ab	2.68 ± 0.04 bc	7.56 ± 0.10 ab	0.47 ± 0.01 a
	8	4.99 ± 0.14 a	2.70 ± 0.05 bc	7.68 ± 0.20 ab	0.49 ± 0.02 a
Korean mint	0	4.68 ± 0.10 a	2.36 ± 0.03 a	7.03 ± 0.13 a	0.65 ± 0.01 a
	0.5	4.34 ± 0.04 b	2.19 ± 0.01 b	6.53 ± 0.05 b	0.60 ± 0.01 ab
	1	3.49 ± 0.09 c	1.90 ± 0.03 c	5.39 ± 0.12 c	0.47 ± 0.02 c
	2	4.24 ± 0.06 b	2.17 ± 0.03 b	6.41 ± 0.09 b	0.58 ± 0.01 b
	4	2.86 ± 0.00 d	1.44 ± 0.01 d	4.30 ± 0.01 d	0.43 ± 0.00 c
	8	2.08 ± 0.06 e	1.21 ± 0.02 e	3.29 ± 0.06 e	0.28 ± 0.01 d
Sweet wormwood	0	4.58 ± 0.08 a	2.77 ± 0.04 b	7.36 ± 0.12 b	0.55 ± 0.01 ab
	0.5	5.00 ± 0.18 a	2.87 ± 0.07 b	7.86 ± 0.25 ab	0.60 ± 0.03 a
	1	4.93 ± 0.16 a	2.89 ± 0.05 ab	7.83 ± 0.21 ab	0.56 ± 0.01 ab
	2	5.11 ± 0.03 a	3.07 ± 0.01 a	8.19 ± 0.04 a	0.65 ± 0.01 a
	4	5.13 ± 0.18 a	2.92 ± 0.04 ab	8.05 ± 0.22 ab	0.64 ± 0.03 a
	8	3.30 ± 0.07 b	1.84 ± 0.03 c	5.14 ± 0.10 c	0.48 ± 0.01 b

Data are represented as mean values ± standard error (*n* = 3). Different letters indicate a significant difference between treatments via Tukey’s HSD test at *p* ≤ 0.05.

## Data Availability

Data are contained within the article.
